# Durable humoral immunity and long-term protection induced by a Crimean-Congo hemorrhagic fever virus replicon particle vaccine in mice

**DOI:** 10.1038/s41541-025-01293-9

**Published:** 2025-11-21

**Authors:** Teresa E. Sorvillo, Elif Karaaslan, Katherine A. Davies, Stephen R. Welch, Florine E. M. Scholte, JoAnn D. Coleman-McCray, Virginia Aida-Ficken, Scott D. Pegan, Éric Bergeron, Joel M. Montgomery, Christina F. Spiropoulou, Jessica R. Spengler

**Affiliations:** 1 Viral Special Pathogens Branch, Division of High-Consequence Pathogens and Pathology, Centers for Disease Control and Prevention, Atlanta, GA USA; 2 Infectious Disease Department, CDC Foundation, Atlanta, GA USA; 3 Zoonotic and Emerging Disease Research Unit, National Bio and Agro-Defense Facility, Agricultural Research Service, United States Department of Agriculture, Manhattan, KS USA; 4 Department of Pathobiology, College of Veterinary Medicine, Auburn University, Auburn, AL USA; 5 Division of Biomedical Sciences, University of California Riverside, Riverside, CA USA

**Keywords:** Vaccines, Antibodies

## Abstract

A Crimean-Congo hemorrhagic fever virus replicon particle vaccine was evaluated for long-term immunity and efficacy in mice. IgG responses persisted up to 18 months, with similar titers across dosing strategies through 12 months. Protective efficacy reached ≥75% at 6 months (prime-only) and up to 12 months (prime-boost). Booster dosing enhanced antibody avidity, effector function, and improved long-term protection. These findings support durable immunity from single or boosted vaccination.

Crimean-Congo hemorrhagic fever virus (CCHFV) is a World Health Organization (WHO) priority pathogen for which there are limited medical countermeasures and no FDA approved vaccines^1^. CCHFV can cause a spectrum of clinical disease including severe hemorrhagic symptoms, with case fatality rates estimated between 4-40%^2^. It has the broadest endemic range of any tickborne virus with thousands of cases estimated to occur each year in highly endemic regions such as Turkey^3,4^.

Vaccine durability is a critical consideration in vaccine development and evaluation. Live-attenuated and virus-like particle (VLP)-based vaccines elicit long-lasting antibody responses that can last for decades or even a lifetime, without booster doses or reactivation of immunological memory^5^. With both platforms, all antigens that are found on the surface of authentic virus are presented to the immune system. A viral replicon particle (VRP) serves as an intermediary to VLP and live-attenuated vaccines. CCHFV VRPs resemble authentic virions, containing genetic material that allows for a single round of replication and translation, but not egress or subsequent cell entry. This non-spreading vaccine platform elicits both humoral and cellular immune responses while maintaining a high safety profile^6–10^.

We have reported single-dose efficacy of VRP vaccines against lethal challenge for several high consequence pathogens: Lassa virus (LASV)^10^, Nipah virus (NiV)^9^ and CCHFV^6^. VRPs could also elicit heterologous protection against diverse virus strains^11,12^ and protect in as little as three days following vaccination^9,13^. While rapid onset of protection is crucial for outbreak response, long-lasting protection is also important. Durable vaccines lessen the need for frequent boosters, increasing cost-effectiveness, decreasing reliance on vaccine compliance, and enhancing the potential to achieve herd immunity and improve epidemic control.

To assess durability of humoral immunity induced by the CCHFV VRP, we quantified anti-nucleoprotein (NP) antibody responses after subcutaneous (SC) vaccination (target dose: 1 × 10^5^ TCID_50_ each) given as a single-dose (prime-only) or two-dose (prime-boost regimen), in which the boost was administered 28 days after the prime, in C57BL/6J mice for up to 18 or 12 months, respectively (Fig. 1A). Anti-NP antibody responses were evaluated because, despite being non-neutralizing, they have been shown to confer protection against CCHF^7,14–16^. In contrast, neutralizing antibodies (directed against the glycoproteins Gc or Gn) have not been consistently correlated with protection^17–23^. Further, a single dose of the CCHFV VRP vaccine platform in rhesus macaques or mice up to 28 and 56 days post-vaccination, respectively, elicited strong IgG responses against NP, with absent or minimally detected antibodies against the glycoproteins (Gc, Gn, GP38)^7,8,24^, consistent with LASV VRP and NiV VRP immunogenicity data^9,25^, indicating a predominance for non-neutralizing anti-NP antibodies elicited by VRP vaccines. Plasma was obtained at designated timepoints following prime-only or prime-boost vaccination (6, 21, 28, or 42 days [D]; and 2, 3, 4, 5, 6, 9, 12, or 18 months [M]), and from unvaccinated age-matched controls (*n* = 10–20 per timepoint/vaccine regimen [5–10 male and 5–10 female]) (Fig. 1A, Table S1). Anti-NP IgG antibody response kinetics including antibody titer, avidity, subclass composition (IgG1, IgG2c), and Fc-mediated effector functions [antibody-dependent complement deposition (ADCD) and antibody-dependent cellular phagocytosis (ADCP)] were quantified at predetermined timepoints after prime-only or prime-boost vaccination. Consistent with previously published data, no sex-based differences in immune responses were detected^8^.

**Fig. 1:**
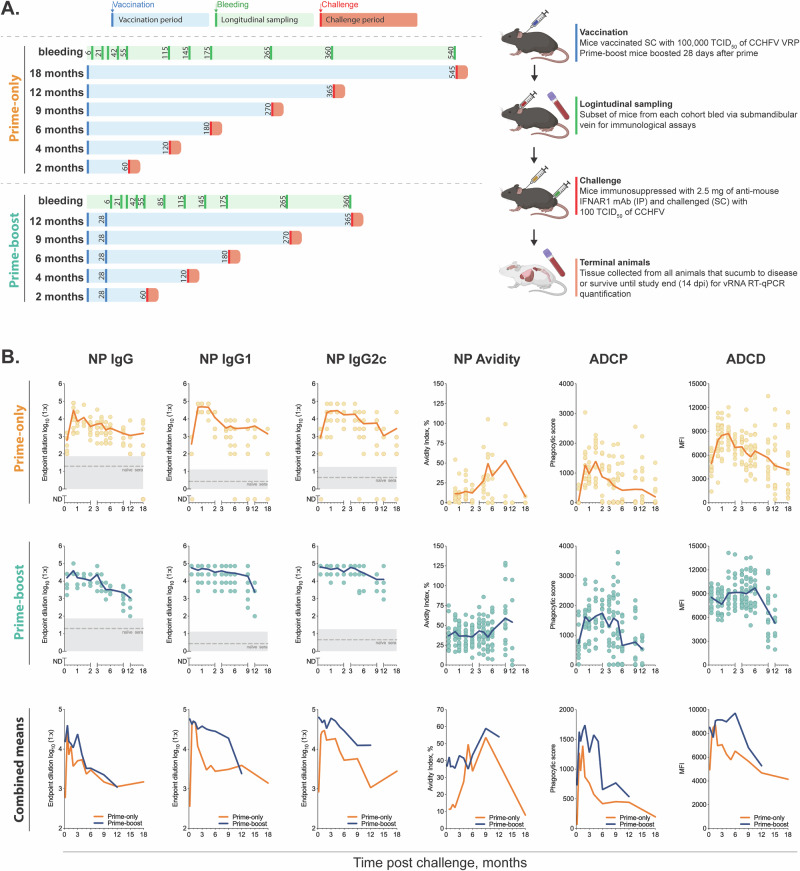
CCHFV viral replicon particle vaccine study design and anti-NP IgG antibody response kinetics. **A** Groups of male and female C57BL/6J mice received either prime-only or prime-boost immunization with 1 × 10^5^ TCID_50_ of CCHFV VRP (subcutaneous [SC]). At designated timepoints following vaccination (prime-only or prime-boost: 6, 21, 28, or 42 days [D]; and 2, 3, 4, 5, 6, 9, 12, or 18 months [M]) whole blood was collected from the submandibular vein (vaccinated and unvaccinated controls: *n* = 10–20 [5–10 male, 5–10 female], each) for assessment of humoral immunity. Lethal challenge studies were conducted with cohorts of vaccinated (prime-only: *n* = 6–16; prime-boost: *n* = 8 per group) and unvaccinated control mice (*n* = 6–12 per group) at a subset of timepoints used to evaluate humoral responses [(2, 4, 6, 9, and 12M (prime-only or prime-boost) and 18M (prime-only)]. On the day of challenge (0 dpi) mice were transiently immunosuppressed with 2.5 mg of anti-mouse IFNAR1 MAR1-5A3 monoclonal antibody (intraperitoneally [IP]), challenged SC with a target dose of 1 × 10^2^ TCID_50_ of CCHFV strain IbAr10200, and followed for up to 14 days post-infection (dpi). **B** Anti-NP IgG antibody response kinetics were quantified and included anti-nucleoprotein (NP) antibody titer, subclass composition (IgG1 and IgG2c), avidity, and the Fc-mediated effector functions antibody-dependent complement deposition (ADCD) and antibody-dependent cellular phagocytosis (ADCP). Each circle represents an individual animal; points are aligned for clarity, and some overlap may occur. The solid line indicates the mean. Where appropriate, the dashed grey line indicates the assay mean from control naïve sera (unvaccinated), and the shaded grey area represents ±1 standard deviation. See Supplemental Figs. 1 and 2 for statistical analyses.

Antibodies were detected (i.e., endpoint dilution significantly elevated compared to unvaccinated controls) in all vaccinated groups at all timepoints for both vaccine regimens (Figs. 1B, S1). Significant differences were detected in IgG titers between prime-only and prime-boost mice at 6D, 21D, 2M, and 3M, but not at long-term timepoints (6M–12M) (Figs. 1B, S1). In the prime-only group, IgG titers peaked between 21D and 42D, then gradually declined from 2 to 6M, returning to early post-vaccination levels (6D) between 9 and 18M (Fig. 1B). In contrast, the prime-boost group exhibited higher titer responses early after boost vaccination, peaking at 21D and remaining elevated through 3M, followed by a gradual decline at 4M and stabilization (no statistical difference) from 5 to 12M (Figs. 1B, S1). Compared to prime-only, the prime-boost group showed an extended high-titer phase early after vaccination (Figs. 1B, S1). Levels of anti-NP avidity, or the strength of binding between antibodies and NP, have been shown to correspond with enhanced VRP vaccine efficacy^7^. Here, infrequent and transient increases in avidity in the prime-only group were observed, with significance detected only at 42D, 4M, and 5M compared to early post-vaccination at 6D (median avidity index: 10–26%) (Figs. 1B, S2). In contrast, the prime-boost regimen elicited higher avidity responses, with a median avidity index of 35–50% from 6D to 9M post-vaccination, sustained across nearly all timepoints except 12M, which still significantly exceeded 12M levels observed in the prime-only group (Figs. 1B, S2), indicating stronger affinity maturation post-boost.

Previously we detected NP specific IgG1, IgG2b, and IgG2c, but not IgG3 in mice sampled up to 56 days post single-dose vaccination^8^. In those studies, both IgG2b and IgG2c, each described to facilitate Fc-mediated effector functions^26^, were upregulated similarly but we found slightly higher titers of 2c. Based on these data, two subclasses of anti-NP antibodies (IgG1, IgG2c) were assessed herein up to 18M. In the prime-only group, 21D, 28D, 42D, and 2M were the only timepoints to show significantly higher IgG1 levels compared to 6D, with 21D to 42D marking the peak IgG1 phase (Figs. 1B, S1). In contrast, the prime-boost group showed no significant drop up to 6M, indicating more sustained titers. Comparable patterns were observed in IgG2c titers between the two vaccine regimens (Figs. 1B, S1). Previously, NP-specific antibodies were shown to possess ADCD and ADCP activity up to 8 weeks post prime-only vaccination and ADCD was shown to be associated with enhanced protective efficacy of the CCHFV VRP vaccine in mice^7,8^. Here, extended follow up of vaccinated mice revealed that antibody effector functions were durable and could be detected in a subset of mice up to the last timepoints assessed, but declined gradually over time, mirroring the waning of total IgG titers (Fig. 1B). While these responses were detected in all mice at earlier timepoints, they were present in only a subset at later timepoints. Importantly, prime-boosted animals had higher ADCD and ADCP function compared to prime-only, despite comparable IgG titers from 6 to 12M (Figs. 1B, S2). A booster increased and stabilized effector function levels for longer with waning seen at 6M versus 3M with prime alone (Figs. 1B, S2). These results collectively highlight the enhanced durability of some important aspects of antibody-mediated protection such as quality and effector function achieved through boosting.

To assess durability of protection, cohorts of VRP vaccinated (prime-only: *n* = 6–16; prime-boost: *n* = 8 per group) and non-vaccinated control mice (*n* = 6–12 per group) were transferred to biosafety level (BSL)-4 high containment laboratories for lethal challenge studies at a subset of timepoints used to evaluate humoral responses [(2, 4, 6, 9, and 12M (prime-only or prime-boost) and 18M (prime-only)] (Fig. 1A, Table S2). On day 0, mice were transiently immunosuppressed (IS mouse model, see methods)^27^, challenged SC with a target dose of 1 × 10^2^ TCID_50_ of CCHFV strain IbAr10200, and followed for up to 14 days post-infection (dpi) (Fig. 1A). The IS mouse model of CCHF was utilized because it allows immunocompetent mice to be vaccinated and develop authentic adaptive immune responses prior to immunosuppression with an anti-IFNAR1 monoclonal antibody (mAb MAR1-5A3) at the time of challenge. All unvaccinated mice succumbed to infection 4–8 dpi (Fig. 2A). Prime vaccination alone conferred high level protection (≥67% survival) at ≤6M, waning to 50, 46, and 21% protection at 9, 12, and 18M, respectively (Fig. 2A). Efficacy levels increased and were longer-lived in mice that received prime-boost vaccination. Protective efficacy was 100% up to 4M post-boost and remained ≥75% up to 12M where 7 of 8 (88%) vaccinated mice survived (Fig. 2A). Tissues [liver, spleen, ovary/testis, cervix/seminal vesicle, kidney, heart, lung, eye and brain] were collected at endpoint for RT-qPCR analyses in all mice (Figs. 2B, S3). Viral RNA was not detected or significantly reduced in tissues from VRP vaccinated survivors (Figs. 2B, S3, Table S3). In a subset of samples vRNA levels were significantly lower in surviving animals receiving prime-boost versus prime-only vaccination (4M: spleen; 9M: liver, eye, brain; 12M: spleen) (Figs. 2B, S3, Table S3). Lower vRNA levels were also detected in the livers of survivors from cohorts challenged earlier after last vaccination compared to survivors that were challenged after longer vaccination periods, indicating that while protective efficacy against severe or lethal disease was maintained, relative control of viral replication decreased over time (Figs. 2B, S3, Table S3).

**Fig. 2:**
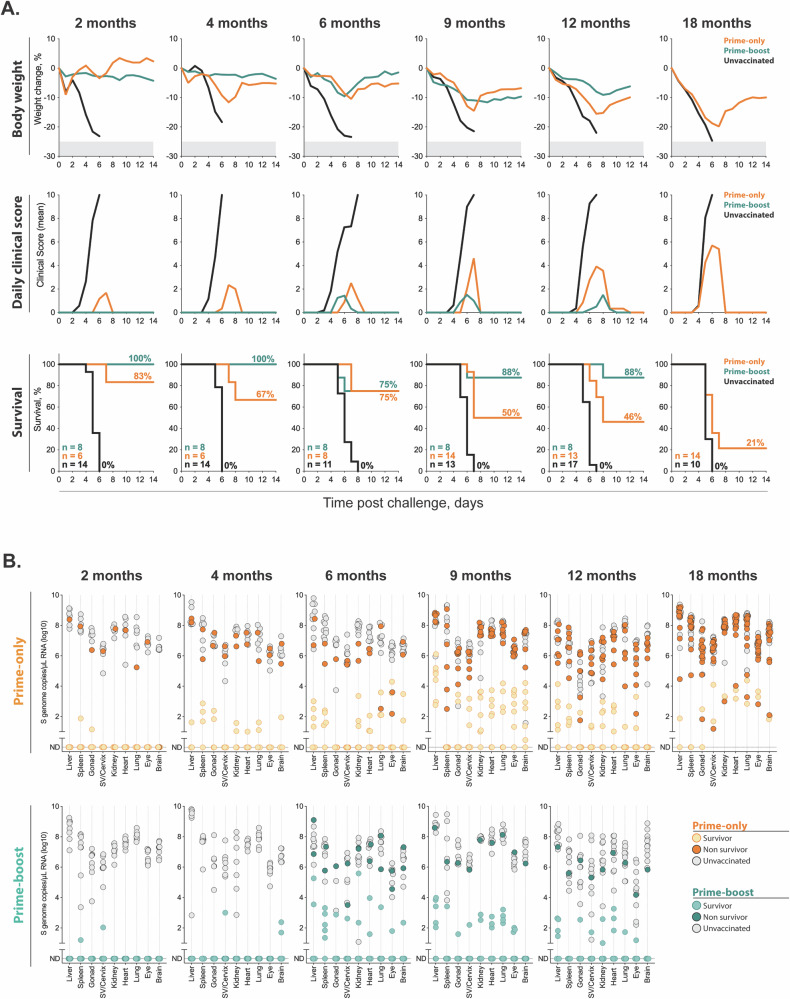
Durability of protection conferred by CCHFV viral replicon particle vaccine in virus challenge studies. **A** Graphs representing mean body weight, daily clinical score, and survival for lethal challenge studies conducted on cohorts of CCHFV VRP vaccinated C57BL/6J mice: prime-only vaccination (orange line); prime-boost vaccination (teal line); and unvaccinated control mice (black line). Mice were transiently immunosuppressed with 2.5 mg of anti-mouse IFNAR1 MAR1-5A3 monoclonal antibody (intraperitoneally, IP), challenged subcutaneously (SC) with a target dose of 1 ×10^2^ TCID_50_ of CCHFV strain IbAr10200 at indicated timepoints after prime-only or prime-boost vaccination and followed for up to 14 days post-infection (dpi). Challenge studies were conducted at a subset of timepoints post final vaccination: 2, 4, 6, 9, and 12 months for prime-only or prime-boost animals; and 18 months for prime-only animals. All unvaccinated mice succumbed to infection at 4–8 dpi. On survival graphs, n represent the animal number per cohort, with total survival per cohort (%) also shown. **B** CCHFV vRNA loads in tissues (liver, spleen, reproductive tissue (ovary or testis and cervix or seminal vesicle), kidney, heart, lung, eye and brain) collected at experimental endpoint were determined by RT-qPCR. Each circle represents an individual animal: grey shaded circles indicate unvaccinated animals; bold shaded circles (orange, prime-only; teal, prime-boost) indicate vaccinated animals that succumbed to infected; and light shaded circles (orange, prime-only; teal, prime-boost) indicate vaccinated animals that survived infection. See Supplemental Table 3 for statistical analyses.

Overall, these data indicate that CCHFV VRP-elicited antibody responses are sustained and correspond to durable protection against lethal disease in the IS mouse model, providing key support for clinical utility of the vaccine. We found that CCHFV VRP in mice elicits sustained IgG titers, avidity, and effector function and improves clinical outcome in a lethal challenge model over a year after vaccination (Fig. 3). Vaccine efficacy studies for prevention of high-consequence low-incidence viruses such as CCHFV have traditionally focused on short vaccination periods with rapidity of protection prioritized. Here, IgG responses were detected up to 18 months post-vaccination, with comparable titers between prime-only and prime-boosted animals. We found that a booster dose improved antibody avidity, as well as Fc-mediated effector function. It also generated responses that were more stable and consistent in magnitude early after boost vaccination compared to prime vaccination alone (Fig. 3). These data are consistent with numerous studies showing that boosting improves vaccine efficacy by increasing and stabilizing antibody titers for longer periods of time as well as improving the quality (avidity) of antibodies through affinity maturation^28,29^. Overall, vaccination improved survival and reduced clinical signs in all cohorts evaluated with prime-boost conferring higher efficacy at later timepoints (Fig. 3).

**Fig. 3:**
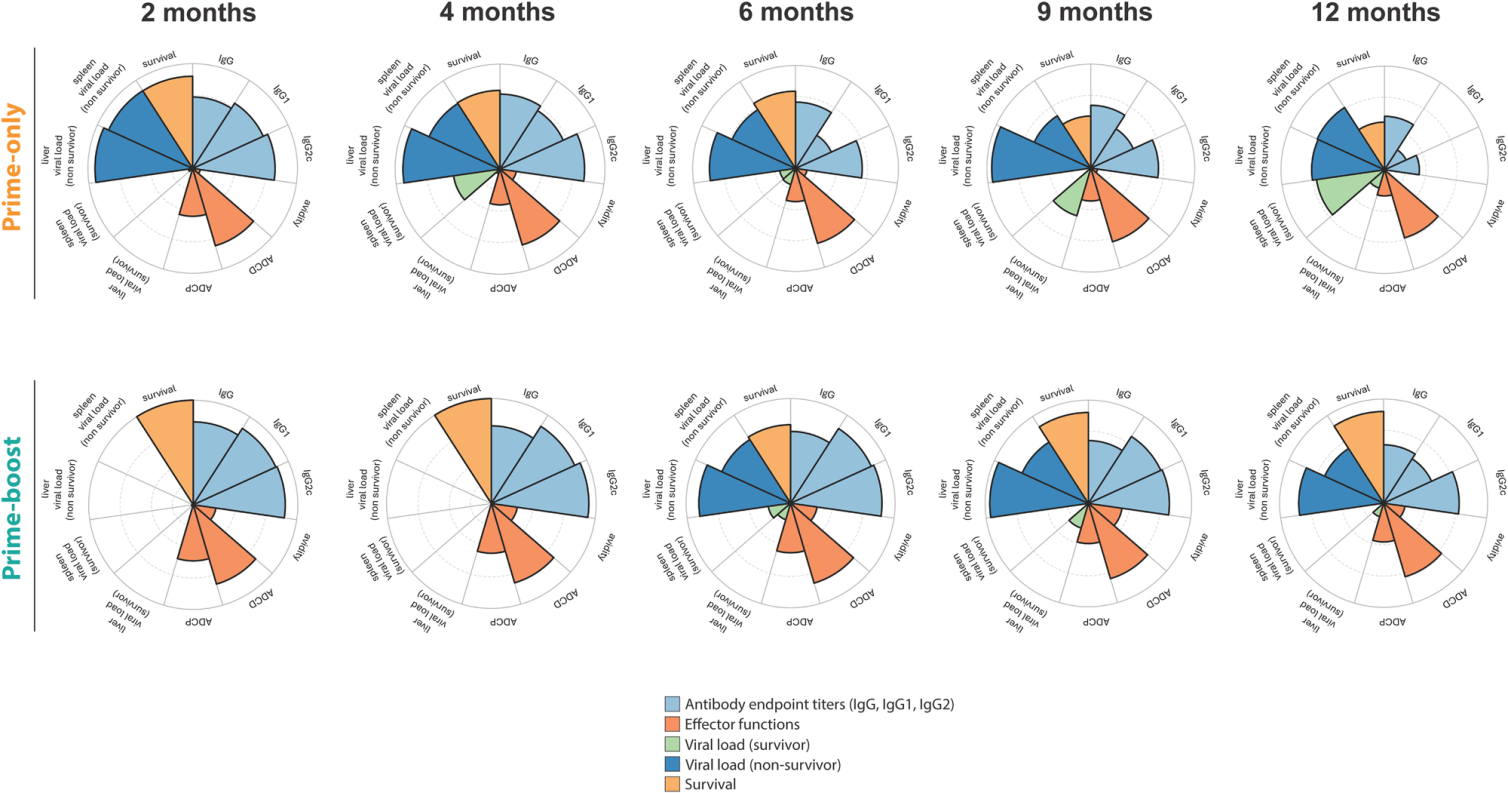
Summary of the kinetics of immune responses, viral load, and survival in mice challenged with CCHFV strain IbAr10200 following two CCHFV VRP vaccination regimens. Nightingale plots illustrate VRP-induced mean antibody titers (IgG, IgG1, IgG2c) and effector functions in mice that received prime-only (top) or prime-boost vaccination (bottom), measured at timepoints corresponding to lethal virus challenge. Also shown are survival rates (%), and viral loads (RNA copy number) in liver and spleen from mice that succumbed (non survivor) or survived (survivor) virus challenge. Wedge size indicates the magnitude of each parameter. Colors represent specific features: light blue, antibody endpoint titers; orange, effector functions; light orange, survival; green, viral load of survivors; dark blue, viral load of non survivors.

Vaccines can provide protection lasting over 20 years, but achieving this level of durability is complex and depends on factors related to the vaccine and vaccination strategy, target pathogen, and individual characteristics of the vaccinee^5^. Achieving optimal vaccine durability also requires knowledge of key correlates of protection which are not yet known for CCHFV. Our studies focused on long-term humoral immunity, but cellular immunity can also play a crucial role in the durability of vaccine protection^30^. Indeed, our previous work in mice demonstrated that NP-specific T cell activation was elicited after CCHFV VRP vaccination^8^. However, our follow up studies demonstrated that T cell responses were not associated with protective efficacy after virus challenge^7^. These studies also showed that NP-specific humoral immunity is key for VRP vaccine efficacy^7^. Future additional studies are warranted to evaluate the durability of T cell-mediated responses and their contribution to long-term protection after CCHFV VRP vaccination.

There remains relatively limited data on durability of protection for viral hemorrhagic fever vaccines. Studies in vivo have evaluated the durability of vesicular stomatitis virus (VSV)-vectored vaccines for Ebola, Sudan, Marburg, Lassa and Nipah viruses, demonstrating that long-term protection (up to 18 months) is achievable^31–35^. However, protection may vary depending on vaccine immunogen, dosing, and challenge virus (homologous versus heterologous). A self-replicating CCHFV RNA vaccine that induces high titers of anti-NP antibodies and T-cell responses against the glycoprotein was evaluated up to 1 year post prime-boost regimen; immune responses and control of viral replication waned over time, but 80% of mice were protected against lethal challenge 1 year post vaccination^36^. Importantly, these data along with our findings support the potential for efficacious and durable platforms to prevent CCHF.

In conclusion, these findings demonstrate that CCHFV VRP vaccination elicits long-lasting antibody responses and confers durable protection against lethal CCHFV challenge in a stringent mouse model, supporting further clinical development. Additionally, we show that the vaccine’s utility for preventing CCHF over extended periods can be enhanced with a booster dose, which conferred sustained immunity and increased efficacy. These findings support ongoing efforts to develop efficacious, clinically applicable vaccines for high-consequence pathogens, where rapid induction of durable protective immunity is critical.

## Methods

### Biosafety and ethics statement

Experiments involving CCHFV were conducted in the BSL-4 laboratory at the Centers for Disease Control and Prevention (CDC; Atlanta, GA, USA). Experiments involving cDNA-encoding viral sequences were performed in accordance with approved Institutional Biosafety Committee protocols. Animal studies were conducted in compliance with the *Guide for the Care and Use of Laboratory Animals* and approved by the CDC Institutional Animal Care and Use Committee (IACUC; #3043, 3102, 3342). The CDC is fully accredited by AAALAC International.

### Virus and cell lines

Recombinant CCHFV IbAr10200 (VirHarv #813730; recIbAr10200; Africa-3 clade) is based on the sequence of a Nigerian tick isolate passaged 9× in suckling mouse brain and 3× in HepG2 cells (GenBank: KJ648914, KJ648915, and KJ648913). Recombinant IbAr10200 was rescued in Huh7 cells and passaged 3× in BSR-T7/5 cells. Vero-E6 cells stably expressing the codon-optimized Oman-98 GPC for vaccine generation were described previously^8^. BSR-T7/5 cells were a kind gift of K.K. Conzelmann (Ludwig-Maximilians-Universität, Munich, Germany). Vero-E6, BSR-T7/5, and Huh7 cells were cultured in DMEM supplemented with fetal calf serum, non-essential amino acids, sodium pyruvate, L-glutamine, penicillin/streptomycin, and puromycin (Vero-E6 Oman-98 GPC cells only). THP-1 cells (ATCC; Cat. No. TIB-202) were cultured in RPMI supplemented with fetal calf serum and antibiotics.

### Vaccine stock production and characterization

CCHFV VRP stocks were generated as previously described^6^. Briefly, CCHFV VRPs were generated by transfecting HuH-7 cells with plasmids encoding CCHFV strain IbAr10200 S and L genomic segments (pT7-S and pT7-L; GenBank: KJ648914 and KJ648913) and strain Oman-98 glycoprotein (pCAGGS-GPC-Oman; GenBank: ALT31693.1) along with plasmids encoding the codon-optimized polymerase (pCAGGS-L), nucleoprotein (pCAGGS-NP), and T7 polymerase (pCAGGS-T7). Supernatants were propagated and quantified via immunofluorescent TCID_50_ assay using Vero-E6 cells stably expressing the codon-optimized Oman-98 GPC; immunostaining was performed using a rabbit polyclonal anti-CCHFV N antibody (1:2500; IBT; Cat. No. 04-0011) followed by a goat anti-rabbit secondary antibody (1:2500; Alexa Flour 488, Invitrogen; Cat. No. A-11008). Wells were scored visually for the presence or absence of fluorescent cells, and TCID_50_ values were calculated using the Reed-Muench method^37^. All vaccine stocks were verified to be mycoplasma free (MycoAlert PLUS detection kit, Lonza LT07), and genomic sequences were confirmed via next-generation sequencing prior to in vivo studies. Backtiter quantification of vaccine inoculum used on day of vaccination (day 0) was performed as described above.

### Longitudinal evaluation of VRP immunogenicity and efficacy in mice

For immunogenicity studies, male and female C57BL/6J mice (The Jackson Laboratory; Strain #000664) were immunized subcutaneously (SC) in the interscapular area with CCHFV VRP (target dose: 1 × 10^5^ TCID_50_ for all vaccinations, whether single or for each dose in the prime-boost regimen); mice were 45 days of age at prime vaccination for both prime-only or prime-boost mice and 73 days of age at boost (prime-boost only; boost interval of 4 weeks). At designated timepoints following prime or boost vaccination (6, 21, 28, or 42 days; and 2, 3, 4, 5, 6, 9, 12, or 18 months) whole blood was collected from the submandibular vein in lithium heparin microtubes (Sarstedt; REF 41.1393.105) of males and females (vaccinated and unvaccinated controls; *n* = 10–20 per timepoint/vaccine regimen [5–10 male and 5–10 female]) using 3–5 mm sterile lancets (Goldenrod) and separated by centrifugation to obtain plasma. For efficacy studies, cohorts of vaccinated (prime-only: *n* = 6–16; prime-boost: *n* = 8 per group) and unvaccinated control mice (*n* = 6–12 per group) were transferred to biosafety level (BSL)-4 high containment laboratories for lethal challenge studies at a subset of timepoints used to evaluate humoral responses [(2, 4, 6, 9, and 12 months (prime or prime-boost) and 18 months (prime only)]. At the time of challenge (day 0) mice were transiently immunosuppressed with 2.5 mg of anti-mouse IFNAR1 monoclonal antibody administered intraperitoneally (MAR1-5A3; Leinco Technologies; Cat. No. I-401, Lot No. 1223L560, 0823L230, 0822L285, and 0323L565). MAR1-5A3 binds to the type I IFN receptor subunit 1, impairing IFN signaling and conferring susceptibility to lethal CCHF disease. After mAb delivery, mice were challenged subcutaneously with a target dose of 1 × 10^2^ TCID_50_ of CCHFV strain IbAr10200 and followed for up to 14 dpi.

### RT-qPCR

RNA was extracted from homogenized tissues including liver, spleen, gonad (testis/ovary), seminal vesicle/cervix, kidney, heart, lung, eye, and brain (homogenized in 1.0 mL of MagMAX lysis buffer). MagMAX Pathogen RNA/DNA kits (Thermo Fisher Scientific; Cat No. 4462359) were used in conjunction with the 96-well ABI MagMAX extraction platform; RNA was eluted into 75 µL of elution buffer. Viral RNA was quantified using a primer/probe set targeting the NP open reading frame of the S genomic segment of CCHFV strain IbAr10200 (forward: 5′-CAG GAC ATG GAC ATA GTG GC-3′; reverse: 5′-ATT GCC CTT GAC GTT GTA GG-3′; probe: 5′-CCC TTG TTG GCA AGC AAT CCC-3′ [all IDT]) using the SuperScript III Platinum One-Step RT-qPCR kit (Thermo Fisher Scientific; Cat No. 11732088). Tissue RNA levels were normalized using the validated reference genes *Ppia* and *Gusb*^38^. Viral RNA copy numbers were quantified via standard curves generated from an RNA standard of known concentration (IDT).

### Serology

For the investigation of anti-NP humoral immune responses, recombinant CCHFV Kosovo Hoti nucleoprotein (GenBank: AW63616.1) was used. The protein expression and purification were performed as previously described^16^. Briefly, the sequence was optimized for bacterial expression and cloned into pET28a by Twist Bioscience. The construct was transformed in *Escherichia coli* BL21 (DE3) strain (Thermo Fisher; Cat. No. EC0114), and the culture was induced with 1 mM isopropyl β-D-1-thiogalactopyranoside. The culture was transferred to 16 °C for overnight incubation, cells were harvested by centrifugation, resuspended in lysis buffer (500 mM NaCl, 20 mM Tris-Cl [pH 7], 0.1% Triton-X, 5% glycerol, 1 mM MgCl_2_, 25 U/mL benzonase), and sonicated. CCHFV NP was purified by nickel affinity chromatography (HisTrap Excel column; Cytiva; Cat. No. 17371205), followed by size exclusion chromatography (SEC; Superdex 200 increase 16/600; Cytiva; Cat. No. 28989335).

Immulon 2HB plates were coated with 100 μL of 500 ng/mL antigen prepared in PBS and incubated overnight at 4 °C. Wells were washed 3× with 300 μL PBS-T (0.1% Tween-20 in PBS) and blocked (5% w/V non-fat dry milk in PBST) for 1 h at room temperature (RT). Following blocking, the buffer was decanted, and 100 μL of mouse plasma prepared in blocking buffer with twofold serial dilutions (range 1:100 to 1:102400) was added to the wells in duplicate. After 1 h incubation at RT, wells were washed 3×, anti-mouse IgG HRP (1:3000, Invitrogen; Cat. No. 61-6520 was added to the wells (100 μL), and plates were incubated for 1 h at RT. Following incubation, wells were washed 3×, and 100 μL TMB Ultra ELISA substrate (Thermo Fisher) was added and incubated for 10 min at RT. The reaction was stopped by adding ELISA stop solution (Thermo Fisher), and optical density was read at 450 nm on a Synergy Neo2 instrument (BioTek) microplate reader. A cut-off value was determined for each plate based on the average absorbance value of negative control wells plus 3 standard deviations. The highest dilutions with a signal above the determined cut-off value were assigned as the endpoint titers. For the subclass IgG ELISA, the assay was performed as described above, and IgG1-specific (Abcam; Cat. No. ab97240), and IgG2c-specific (Abcam; Cat. No. ab97255) anti-mouse antibodies conjugated to HRP were used in a 1:3000 dilution. The results were represented as the endpoint titer of each sample.

The avidity ELISA assay was performed as described above with an additional treatment step using a chaotropic agent. Briefly, plasma samples from individual animals were prepared in 3-fold dilution series in 2 replicates. Plates were incubated for 1 h as described above and washed 3× with PBST. Then, 200 µL/well PBS was added to one replicate (untreated), while 200 µL/well 6 M urea was added to the other (treated). After 10 min incubation at RT, plates were washed 3× times with PBST, and the rest of the assay was completed as described for IgG ELISA. The area under the curve (AUC) was calculated for both untreated and treated replicates, and the avidity index (Fig. 1B, NP Avidity) was determined by dividing the AUC of the urea-treated wells by that of the PBS-treated wells, and then multiplying by 100^16,39^.

### Fc-mediated effector function

Antibody-dependent complement deposition (ADCD) and antibody-dependent cellular phagocytosis (ADCP) assays were adapted from previously reported methods^40,41^. Recombinant CCHFV NP generated based on strain Hoti was biotinylated (EZ-Link™ Sulfo-NHS-LC-Biotinylation Kit, Thermo Fisher; Cat. No. 21435) and coupled to fluorescent red or green neutravidin microspheres (Thermo Fisher; Cat. No. F8775/F8776). Antigen-coated beads were incubated 2 h at 37 °C with heat-inactivated (30 min at 56 °C) NHP plasma in sodium citrate. For ADCD assays, guinea pig complement (Cedarlane; Cat. No. CL4051) diluted in gelatin veronal buffer (CompTech; Cat. No. B102) was added and incubated 15 min at 37 °C. Beads were washed and incubated for 15 min at room temperature with FITC-conjugated anti-guinea pig complement C3 (MP Biomedicals; Cat. No. 0855385). For ADCP assays, beads were incubated overnight at 37 °C with 2 × 10^4^ THP-1 cells per well. Beads and cells were analyzed on a Guava cytometer. Fold ADCD activation was calculated based on the pre-vaccination (day 0) plasma samples. Phagocytic score was calculated by multiplying the percentage of bead-positive cells by the overall median fluorescence intensity.

### Statistical analysis

Normality of the data analyzed using Shapiro-Wilk test and statistical significance was determined by the two-tailed Kruskal-Wallis for IgG, IgG1, IgG2c, and avidity and ordinary one-way ANOVA for ADCD, ADCP. The Benjamini, Krieger & Yekutieli method applied for multiple pairwise comparisons to control false-discovery rate. The Nightingale plots were generated from log-transformed data for IgG, IgG1, IgG2c, ADCD, ADCP, and tissue viral load, and scaled data. Multiple Mann-Whitney tests were performed to compare vRNA tissue levels between groups (prime, prime-boost, and unvaccinated) and timepoints. All statistical analyses were performed using GraphPad.

### Data visualization

All graphs were generated using GraphPad Prism version 10.2.3. Nightingale plots were generated using RStudio 4.4.0. Figure 1A was created with BioRender.com.

## Supplementary information

Supplemetary Figures

Supplemetary Tables

## Data Availability

Data are available within the article and supplementary material, and from the corresponding author upon reasonable request.

## References

[1] WHO. WHO | List of Blueprint priority diseases. *WHO* (2018).

[2] Ergönül, Ö Crimean-Congo haemorrhagic fever. *Lancet Infect. Dis.* **6**, 203–214 (2006). 16554245 10.1016/S1473-3099(06)70435-2 PMC7185836

[3] Bodur, H., Akinci, E., Ascioglu, S., Öngürü, P. & Uyar, Y. Subclinical infections with Crimean-Congo hemorrhagic fever virus, Turkey. *Emerg. Infect. Dis.* **18**, 640–642 (2012). 22469474 10.3201/eid1804.111374 PMC3309668

[4] Bente, D. A. et al. Crimean-Congo hemorrhagic fever: history, epidemiology, pathogenesis, clinical syndrome and genetic diversity. *Antivir. Res* **100**, 159–189 (2013). 23906741 10.1016/j.antiviral.2013.07.006

[5] Vashishtha, V. M. & Kumar, P. The durability of vaccine-induced protection: an overview. *Expert Rev. Vaccines* **23**, 389–408 (2024). 38488132 10.1080/14760584.2024.2331065

[6] Scholte, F. E. M. et al. Single-dose replicon particle vaccine provides complete protection against Crimean-Congo hemorrhagic fever virus in mice. *Emerg. Microbes Infect.* **8**, 575–578 (2019). 30947619 10.1080/22221751.2019.1601030 PMC6455139

[7] Sorvillo, T. E. et al. Replicon particle vaccination induces non-neutralizing anti-nucleoprotein antibody-mediated control of Crimean-Congo hemorrhagic fever virus. *NPJ Vaccines* **9**, 88 (2024). 38782933 10.1038/s41541-024-00877-1 PMC11116556

[8] Scholte, F. E. M. et al. Vaccination with the Crimean-Congo hemorrhagic fever virus viral replicon vaccine induces NP-based T-cell activation and antibodies possessing Fc-mediated effector functions. *Front. Cell Infect Microbiol.* **13**, 1233148 (2023). 10.3389/fcimb.2023.1233148 PMC10475602 37671145

[9] Welch, S. R. et al. Single-dose mucosal replicon-particle vaccine protects against lethal Nipah virus infection up to 3 days after vaccination. *Sci. Adv* **9**, eadh4057 (2023). 10.1126/sciadv.adh4057 PMC10403222 37540755

[10] Kainulainen, M. H. et al. Use of a scalable replicon-particle vaccine to protect against lethal Lassa virus infection in the guinea pig model. *J. Infect. Dis.* **217**, 1957–1966 (2018). 29800368 10.1093/infdis/jiy123 PMC6086598

[11] Spengler, J. R. et al. Heterologous protection against Crimean-Congo hemorrhagic fever in mice after a single dose of replicon particle vaccine. *Antivir. Res* **170**, 104573 (2019). 31377243 10.1016/j.antiviral.2019.104573 PMC6773275

[12] Spengler, J. R. et al. Lassa virus replicon particle vaccine protects strain 13/N guinea pigs against challenge with geographically and genetically diverse viral strains. *J. Infect. Dis.* **226**, 1545–1550 (2022). 10.1093/infdis/jiac028 PMC11268724 35099012

[13] Spengler, J. R. et al. Viral replicon particles protect IFNAR-/- mice against lethal Crimean-Congo hemorrhagic fever virus challenge three days after vaccination. *Antivir. Res.* **191**, 105090 (2021). 34044061 10.1016/j.antiviral.2021.105090 PMC9250103

[14] Garrison, A. R. et al. Nucleocapsid protein-specific monoclonal antibodies protect mice against Crimean-Congo hemorrhagic fever virus. *Nat. Commun.* **15**, 1722 (2024). 38409240 10.1038/s41467-024-46110-4 PMC10897337

[15] Leventhal, S. S. et al. Antibodies targeting the Crimean-Congo Hemorrhagic fever virus nucleoprotein protect via TRIM21. *Nat. Commun.* **15**, 9236 (2024). 39455551 10.1038/s41467-024-53362-7 PMC11511847

[16] Karaaslan, E. et al. Crimean Congo hemorrhagic fever virus nucleoprotein and GP38 subunit vaccine combination prevents morbidity in mice. *NPJ Vaccines* **9**, 148 (2024). 39143104 10.1038/s41541-024-00931-y PMC11324950

[17] Mousavi-Jazi, M., Karlberg, H., Papa, A., Christova, I. & Mirazimi, A. Healthy individuals’ immune response to the Bulgarian Crimean-Congo hemorrhagic fever virus vaccine. *Vaccine* **30**, 6225–6229 (2012). 22902680 10.1016/j.vaccine.2012.08.003

[18] Canakoglu, N. et al. Immunization of Knock-Out α/β interferon receptor mice against high lethal dose of Crimean-Congo hemorrhagic fever virus with a cell culture based vaccine. *PLoS Negl. Trop. Dis.* **9**, e0003579 (2015). 10.1371/journal.pntd.0003579 PMC4356576 25760444

[19] Kortekaas, J. et al. Crimean-Congo hemorrhagic fever virus subunit vaccines induce high levels of neutralizing antibodies but no protection in STAT1 knockout mice. *Vector-Borne Zoonotic Dis.* **15**, 759–764 (2015). 26684523 10.1089/vbz.2015.1855 PMC7643766

[20] Garrison, A. R. et al. A DNA vaccine for Crimean-Congo hemorrhagic fever protects against disease and death in two lethal mouse models. *PLoS Negl. Trop. Dis.* **11**, e0005908 (2017). 28922426 10.1371/journal.pntd.0005908 PMC5619839

[21] Hawman, D. W. et al. Accelerated DNA vaccine regimen provides protection against Crimean-Congo hemorrhagic fever virus challenge in a macaque model. *Mol. Ther.*10.1016/j.ymthe.2022.09.016 (2022). 10.1016/j.ymthe.2022.09.016 PMC9931546 36184852

[22] Hawman, D. W. et al. A DNA-based vaccine protects against Crimean-Congo haemorrhagic fever virus disease in a Cynomolgus macaque model. *Nat. Microbiol.* **6**, 187–195 (2021). 33257849 10.1038/s41564-020-00815-6 PMC7854975

[23] Papa, A., Pappa, S., Panayotova, E., Papadopoulou, E. & Christova, I. Molecular epidemiology of Crimean-Congo hemorrhagic fever in Bulgaria-An update. *J. Med. Virol.* **88**, 769–773 (2016). 26455333 10.1002/jmv.24400

[24] Kleymann, A. et al. Crimean-Congo hemorrhagic fever virus replicon particle vaccine is safe and elicits functional, non-neutralizing anti-nucleoprotein antibodies and T cell activation in rhesus macaques. *Antivir. Res.* **233**, 106045 (2025). 39626793 10.1016/j.antiviral.2024.106045 PMC11871586

[25] Kainulainen, M. H. et al. Protection from lethal Lassa disease can be achieved both before and after virus exposure by administration of single-cycle replicating Lassa virus replicon particles. *J. Infect. Dis.*10.1093/infdis/jiz284 (2019). 10.1093/infdis/jiz284 PMC6744324 31152662

[26] Collins, A. M. IgG subclass co-expression brings harmony to the quartet model of murine IgG function. *Immunol. Cell Biol.* **94**, 949–954 (2016). 27502143 10.1038/icb.2016.65

[27] Sorvillo, T. E. et al. Inflammation associated with monocyte/macrophage activation and recruitment corresponds with lethal outcome in a mouse model of Crimean-Congo haemorrhagic fever. *Emerg. Microbes Infect.* **13**, 2427782 (2024). 39513496 10.1080/22221751.2024.2427782 PMC11578417

[28] Pitisuttithum, P. et al. Pertussis immunity 5 years after booster vaccination with recombinant pertussis vaccines. *JAMA Netw. Open* **7**, e2449182 (2024). 39636641 10.1001/jamanetworkopen.2024.49182 PMC11621984

[29] Gilboa, M. et al. Durability of immune response after COVID-19 booster vaccination and association with COVID-19 Omicron infection. *JAMA Netw. Open* **5**, e2231778 (2022). 36107426 10.1001/jamanetworkopen.2022.31778 PMC9478782

[30] Sun, L., Su, Y., Jiao, A., Wang, X. & Zhang, B. T cells in health and disease. *Signal Transduct. Target Ther.* **8**, 235 (2023). 37332039 10.1038/s41392-023-01471-y PMC10277291

[31] Wong, G. et al. Immunization with vesicular stomatitis virus vaccine expressing the Ebola glycoprotein provides sustained long-term protection in rodents. *Vaccine* **32**, 5722–5729 (2014). 25173474 10.1016/j.vaccine.2014.08.028 PMC7115511

[32] Marzi, A. et al. Species-specific immunogenicity and protective efficacy of a vesicular stomatitis virus-based Sudan virus vaccine: a challenge study in macaques. *Lancet Microbe* **4**, e171–e178 (2023). 36739878 10.1016/S2666-5247(23)00001-0 PMC10010116

[33] Mire, C. E. et al. Durability of a vesicular stomatitis virus-based marburg virus vaccine in nonhuman primates. *PLoS ONE* **9**, e94355 (2014). 24759889 10.1371/journal.pone.0094355 PMC3997383

[34] Stein, D. R. et al. A recombinant vesicular stomatitis-based Lassa fever vaccine elicits rapid and long-term protection from lethal Lassa virus infection in guinea pigs. *NPJ Vaccines* **4**, 8 (2019). 30774999 10.1038/s41541-019-0104-x PMC6368541

[35] Woolsey, C. et al. Recombinant vesicular stomatitis virus-vectored vaccine induces long-lasting immunity against Nipah virus disease. *J. Clin. Investig.* **133**, e164946 (2023). 36445779 10.1172/JCI164946 PMC9888376

[36] Leventhal, S. S. et al. Replicating RNA vaccine confers durable immunity against Crimean Congo hemorrhagic fever virus challenge in mice. *NPJ Vaccines* **9**, 249 (2024). 39702459 10.1038/s41541-024-01045-1 PMC11659298

[37] Reed, L. J. & Muench, H. A simple method for estimating fifty percent endpoints. *Am. J. Epidemiol.* **27**, 493–497 (1938).

[38] Davies, K. A. et al. Optimal reference genes for RNA tissue analysis in small animal models of hemorrhagic fever viruses. *Sci. Rep.* **13**, 19384 (2023). 37938597 10.1038/s41598-023-45740-w PMC10632498

[39] Almanzar, G., Ottensmeier, B., Liese, J. & Prelog, M. Assessment of IgG avidity against pertussis toxin and filamentous hemagglutinin via an adapted enzyme-linked immunosorbent assay (ELISA) using ammonium thiocyanate. *J. Immunol. Methods* **387**, 36–42 (2013). 23022630 10.1016/j.jim.2012.09.008

[40] Fischinger, S. et al. A high-throughput, bead-based, antigen-specific assay to assess the ability of antibodies to induce complement activation. *J. Immunol. Methods* **473**, 112630 (2019). 31301278 10.1016/j.jim.2019.07.002 PMC6722412

[41] Butler, A. L., Fallon, J. K. & Alter, G. A sample-sparing multiplexed ADCP Assay. *Front. Immunol.* **10**, 1851 (2019). 31456799 10.3389/fimmu.2019.01851 PMC6700248

