# Supplementary material for: Durable humoral immunity and long-term protection induced by a Crimean-Congo hemorrhagic fever virus replicon particle vaccine in mice
Source: NPJ Vaccines. 2025 Nov 21;10:244. doi: 10.1038/s41541-025-01293-9 (PMC12639013; doi:10.1038/s41541-025-01293-9)
Supplement: Supplementary file 1 — Supplemetary Figures [file 41541_2025_1293_MOESM1_ESM.pdf]

# Durable humoral immunity and long-term protection induced by a Crimean-Congo hemorrhagic fever virus replicon particle vaccine in mice

Teresa E. Sorvillo[1,2]^, Elif Karaaslan[1]^, Katherine A. Davies[1,3], Stephen R. Welch[1], Florine E.M. Scholte[1], JoAnn D. Coleman-McCray[1], Virginia Aida-Ficken[1,4], Scott D. Pegan[5], Éric Bergeron[1], Joel M. Montgomery[1], Christina F. Spiropoulou[1], Jessica R. Spengler[1]*

**Affiliations:**

[1]Viral Special Pathogens Branch, Division of High Consequence Pathogens and Pathology, Centers for Disease Control and Prevention, Atlanta, GA, USA

[2]Infectious Disease Department, CDC Foundation, Atlanta, GA, USA

[3]Zoonotic and Emerging Disease Research Unit, National Bio and Agro-Defense Facility, Agricultural Research Service, United States Department of Agriculture, Manhattan, KS, USA

[4]Department of Pathobiology, College of Veterinary Medicine, Auburn University, Auburn, AL, USA

[5]Division of Biomedical Sciences, University of California Riverside, Riverside, CA, USA

^Contributed equally.

**Supplementary Figure 1.** Statistical significance of IgG, IgG1, and IgG2c antibody titers over time and between prime and prime-boost vaccine regimens.

**Supplementary Figure 2.** Statistical significance of antibody avidity, ADCD, and ADCP values over time and between prime and prime-boost vaccine regimens.

**Supplementary Figure 3.** Viral load (vRNA) in tissues from prime-only and prime-boost vaccinated mice after CCHFV challenge.

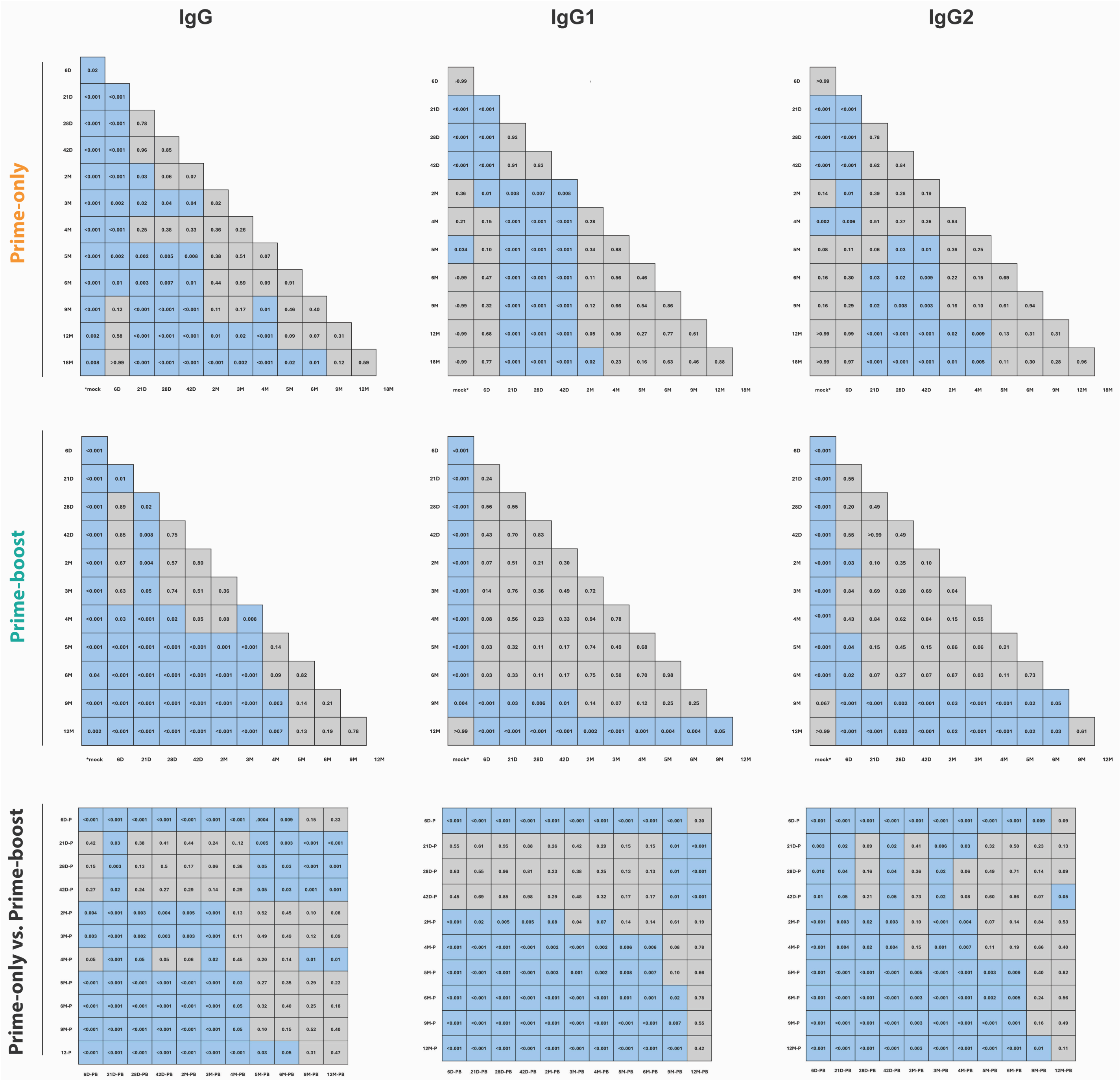


**Supplementary Figure 1. Statistical significance of IgG, IgG1, and IgG2c antibody titers over time and between prime and prime-boost vaccine regimens.** The multiple comparison heatmap displays the p value of immune parameters at each time point for (A) prime-only, (B) prime-boost, and (C) the comparison between prime-only (P) and prime-boost (PB) vaccination regimens, across IgG, IgG1, IgG2c. Blue indicates statistical significance (p<0.05), while gray indicates non-significance (p>0.05). Exact p-values were determined by the two-tailed Kruskal-Wallis test, using the Benjamini, Krieger, and Yekutieli method to control the false discovery rate. Mock indicates values for unvaccinated controls.

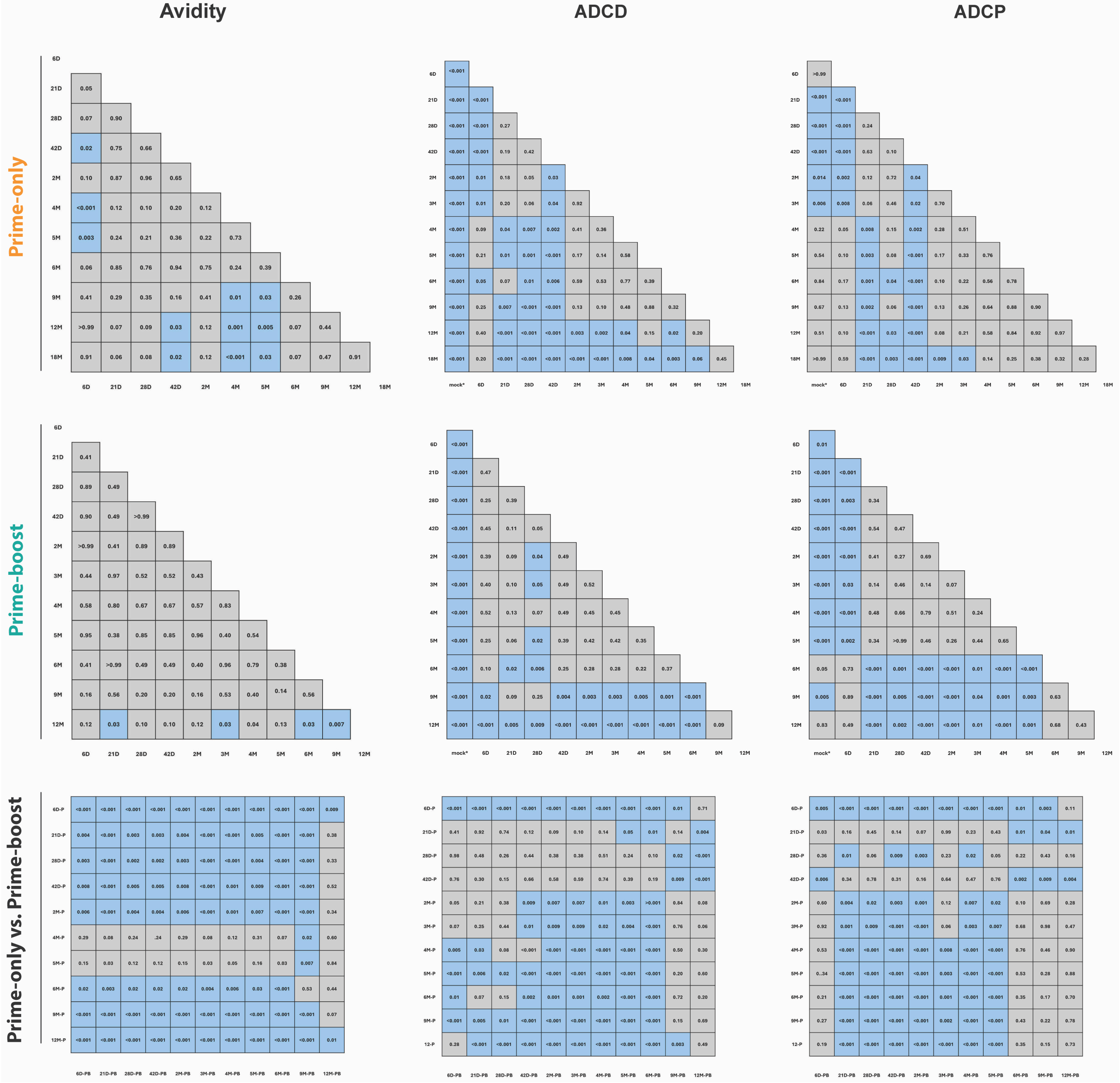


**Supplementary Figure 2. Statistical significance of antibody avidity, ADCD, and ADCP values over time and between prime and prime-boost vaccine regimens.** The multiple comparison heatmap displays the p value of immune parameters at each time point for (A) prime-only, (B) prime-boost, and (C) the comparison between prime-only (P) and prime-boost (PB) vaccination regimens, across avidity, ADCD, and ADCP. Blue indicates statistical significance ($p<0.05$), while gray indicates non-significance ($p>0.05$). Exact p-values were determined by the two-tailed Kruskal-Wallis test for avidity and ordinary one-way ANOVA for ADCD, ADCP, using the Benjamini, Krieger, and Yekutieli method to control the false discovery rate. Mock indicates values for unvaccinated controls.

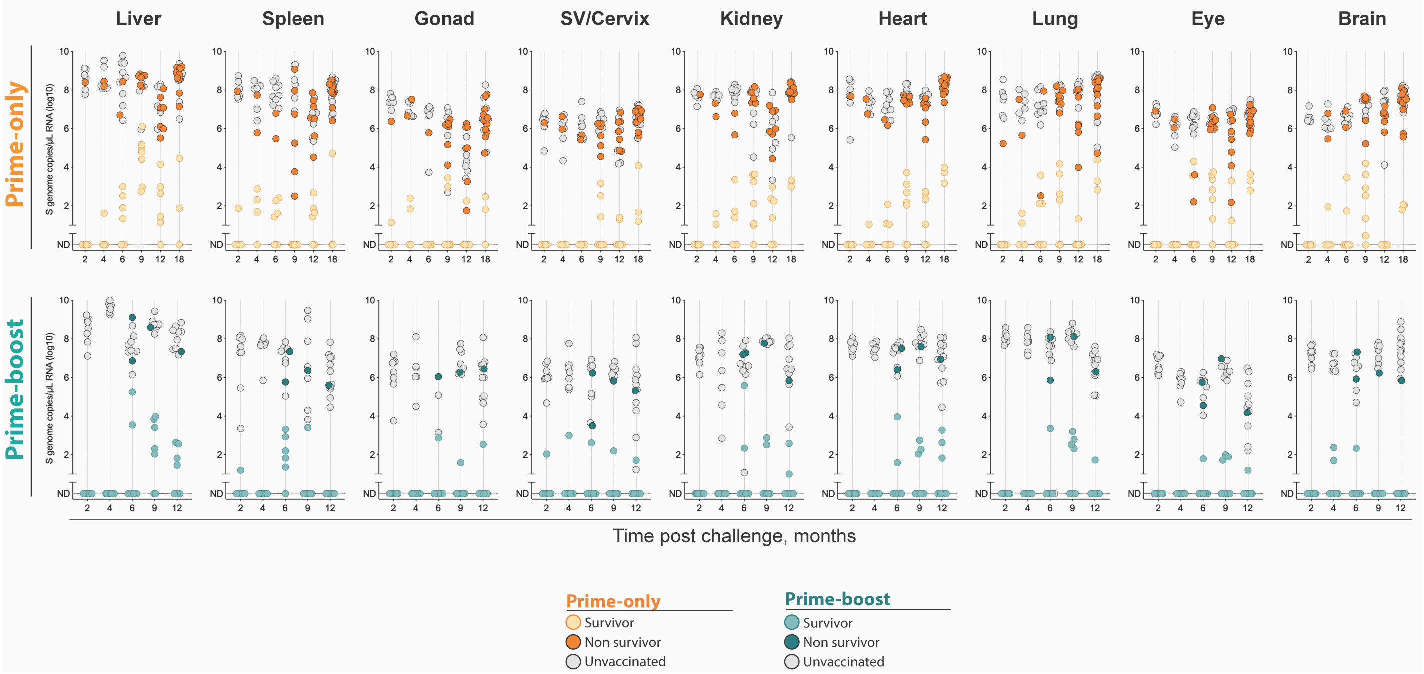


**Supplementary Figure 3. Viral load (vRNA) in tissues from prime-only and prime-boost vaccinated mice after CCHFV challenge.** CCHF vRNA loads in tissues (liver, spleen, reproductive tissue [ovary or testis and cervix or seminal vesicle], kidney, heart, lung, eye and brain) collected at experimental endpoint were determined by RT-qPCR. Each circle represents an individual animal: grey shaded circles indicate unvaccinated animals; bold shaded circles (orange, prime-only; teal, prime-boost) indicate vaccinated animals that succumbed to infected; and light shaded circles (orange, prime-only; teal, prime-boost) indicate vaccinated animals that survived infection. See Supplemental Table 3 for statistical analyses.
